# Interphase Nucleo-Cytoplasmic Shuttling and Localization of SIRT2 during Mitosis

**DOI:** 10.1371/journal.pone.0000784

**Published:** 2007-08-29

**Authors:** Brian J. North, Eric Verdin

**Affiliations:** Gladstone Institute of Virology and Immunology, University of California at San Francisco, California, United States of America; Institute for Research in Biomedicine, Spain

## Abstract

The human NAD+-dependent protein deacetylase SIRT2 resides predominantly in the cytoplasm where it functions as a tubulin deacetylase. Here we report that SIRT2 maintains a largely cytoplasmic localization during interphase by active nuclear export in a Crm1-dependent manner. We identified a functional, leptomycin B-sensitive, nuclear export signal sequence within SIRT2. During the cell cycle, SIRT2 becomes enriched in the nucleus and is associated with mitotic structures, beginning with the centrosome during prophase, the mitotic spindle during metaphase, and the midbody during cytokinesis. Cells overexpressing wild-type or a catalytically inactive SIRT2 exhibit an increase in multinucleated cells. The findings suggest a novel mechanism of regulating SIRT2 function by nucleo-cytoplasmic shuttling, as well as a role for SIRT2 in the nucleus during interphase and throughout mitosis.

## Introduction

The seven human class III histone deacetylases (HDACs) are homologs of the yeast Sir2 protein and are termed sirtuins or SIRT proteins (SIRT1-7) [1]–[3]. Class III HDACs are different from class I and II HDACs in many respects, such as the structure of their catalytic domains [3], [4] and the nature of their small-molecule inhibitors [5], [6]. Furthermore, deacetylation by class III HDACs depends on the co-factor NAD+, which is cleaved during the deacetylation reaction. SIRT1, 2, 3 and 5 function as deacetylases on a histone peptide substrate, but no deacetylation activity has been observed for SIRT4, 6, or 7 [7]. Sirtuins also possess an ADP-ribosyltransferase activity, that is unique to the class III HDACs [1], [8]–[11].

Human sirtuins exhibit distinctive subcellular localizations. SIRT1 and SIRT6 are localized in the nucleus, and SIRT7 is found exclusively in nucleoli [12]. However, in pancreatic ß cells, SIRT1 is localized in the cytoplasm and excluded from the nucleus [13]. The mechanism that regulates the subcellular distribution of SIRT1 in this context remains elusive. SIRT2 often appears distinctly localized in the cytoplasm [7], [12], [14], [15]. SIRT3, SIRT4 and SIRT5 are all localized in the mitochondria; however, only SIRT3 and SIRT4 have been demonstrated to be imported into the mitochondrial matrix [12], [16], [17].

A complex sorting network recognizes specific peptide signal sequences in proteins and controls the proper targeting of various proteins to the cell membrane and organelles such as the mitochondria, endoplasmic reticulum and Golgi. Additionally, many proteins have distinct functions in the nucleus or in the cytoplasm, and specific signals exist for localization to these compartments as well. A network of importin and exportin proteins regulates protein trafficking between the nucleus and cytoplasm. The nuclear import machinery recognizes peptide sequences made up primarily of basic amino acids termed nuclear localization signal (NLS) sequences [18]. Likewise, nuclear export machinery is required to maintain proteins containing nuclear export signal (NES) sequences in the cytoplasm [18], [19]. The nuclear import and export machinery is coupled to the regulation of RAN-GTP hydrolysis. A concentration gradient of RAN-GTP and RAN-GDP between the nucleus and cytoplasm is established through the localization of the RAN-GEF RCC1 tethered to chromatin in the nucleus and Ran-GAP in the cytoplasm (reviewed in [20]).

SIRT2 is predominantly cytoplasmic, but has also been observed in the nucleus [7], [21]. Furthermore, its role in controlling cell-cycle progression during mitosis suggests that SIRT2 may be localized to mitotic structures [22], [23]. In this study, we analyzed interphase nuclear-cytoplasmic shuttling of SIRT2 and characterized its localization throughout the mitosis.

## Materials and Methods

### Tissue Culture

293T and HeLa cells were from American Type Culture Collection (ATCC) and grown in Dulbecco's modified Eagle's medium (DMEM, Mediatech, Herndon, VA) supplemented with 10% fetal bovine serum (Gemini Bio-products, Woodland, CA) in the presence of penicillin, streptomycin and 2 mM L-glutamine (Gibco Invitrogen Corp., Carlsbad, CA).

### Plasmids and Mutagenesis

Human SIRT2 full-length and deletion constructs were cloned into pEGFP-C1 vector (Clontech) and into a derivative of the pcDNA3.1(+) backbone (HA vector) by standard PCR-based strategies. Site-directed mutagenesis for SIRT2 constructs was performed with the QuikChange Site-Directed Mutagenesis Kit (Stratagene, La Jolla, CA) as recommended. FLAG-tagged SIRT2 was previously described [7], fibrillarin-GFP was a kind gift from Dr. Tom Mistelli [24], [25], and Myc-epitope-tagged Aurora A and Auroa B were a kind gift from Dr. Hongtao Yu [26].

### Transient Transfections and Immunoprecipitations

293T cells were transfected by the calcium phosphate DNA precipitation method and lysed 48 h after transfection in low-stringency lysis buffer (50 mM Tris-HCl, pH 7.5, 0.5 mM EDTA, 0.5% NP-40, 150 mM NaCl) in the presence of protease inhibitor cocktail (Complete, Roche Molecular Biochemicals, Indianapolis, IN). FLAG-tagged proteins were immunoprecipitated with anti-FLAG M2 agarose affinity gel (Sigma, St. Louis, MO), for 2 h at 4°C from 1 mg of total cell lysate measured by the *Dc* Protein Assay Kit (Bio-Rad, Hercules, California). Immunoprecipitated material was washed 3 times for 15 min each in low-stringency lysis buffer.

### Western Blotting

Samples were separated on 10% SDS-polyacrylamide gels and transferred to Hybond ECL nitrocellulose membrane (Amersham Pharmacia Biotech). Membranes were blocked with 5% blocking reagent (Bio-Rad) in TBS-Tween (10 mM Tris, pH 7.5, 150 mM NaCl, and 0.1% Tween-20), and incubated with mouse monoclonal anti-GFP (Clontech) diluted 1∶2000, rabbit polyclonal anti-FLAG (Sigma) and anti-Myc (Santa Cruz Biotechnology) were both diluted 1∶5000. Secondary detection was performed using horseradish peroxidase-coupled sheep anti-mouse IgG or anti-rabbit IgG (both from Pierce) and ECL western blotting detection system (Amersham Pharmacia Biotech).

### Immunofluorescence Microscopy

HeLa or U2OS cells grown on coverslips were transfected with Lipofectamine (Gibco InVitrogen) according to the manufacturer's protocol. Cells on coverslips were washed twice in PBS for 10 min, fixed in 4% paraformaldehyde (EMS, Ft. Washington, PA) for 10 min, and permeabilized in 0.5% Triton-X-100 in PBS for 10 min. After 3 washes for 10 min each in PBS, cells were incubated in 10% BSA for 10 min and then incubated for 1 h with anti-FLAG diluted 1∶5000 in PBS+0.1% Tween-20 or Anti-γ-tubulin-Cy3 (Sigma). Cells were washed three times for 10 min in PBS+0.1% Tween-20, followed by incubation with goat anti-mouse IgG (Fc specific) TRITC-conjugated secondary antibody (Sigma) diluted 1:100 in PBS+0.1% Tween-20. Cells were then incubated in 20 µg/ml DAPI for 5 min, washed 3 times for 10 min each in PBS and once briefly in ddH2O, and mounted on slides with Gel Mount (Biomeda,Foster City, CA). Confocal images were acquired by laser-scanning confocal microscopy with an Olympus BX60 microscope equipped with a Radiance 2000 confocal setup (Bio-Rad). For cell count experiments, six random fields were visualized, from which at least 200 cells were counted.

For leptomycin B and cytokinesis experiments, cells were transfected with GFP-SIRT2, and 24 h later treated with and without LMB (Sigma) and incubated for the times indicated or for 16 h. Cells were washed and fixed as described above, and images acquired by confocal microscopy. Cell counts were performed on greater than 200 transfected cells, and results shown are a representative of three independent experiments.

For indirect immunofluorescence of HeLa cells for endogenous proteins, cells were grown on coverslips for 16 h, followed by processing for immunofluorescence as described above. Chicken Anti-SIRT2 was diluted 1∶10. rabbit anti-aurora A and aurora B (Abcam) were diluted 1∶1000, and anti-Ac-tubulin 6-11B-1 (Sigma) was diluted 1∶500. Secondary detection was performed with anti-mouse-TRITC (Sigma), anti-chicken-Cy2 (Jackson Immuno) and anti-rabbit-Cy5 (Jackson Immuno), all diluted 1:500.

## Results

### Nucleo-Cytoplasmic Shuttling of SIRT2

**Figure 1:**
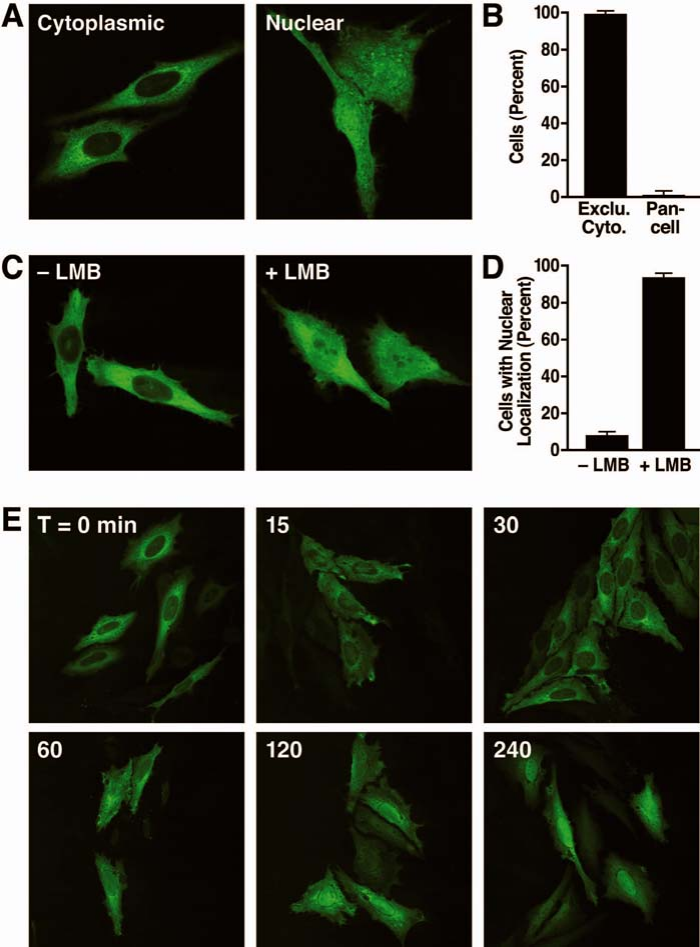
Cytoplasmic localization of SIRT2 is dependent on constitutive nuclear export. (A) HeLa cells were transfected with GFP-SIRT2 and analyzed by immunofluorescence for SIRT2 localization. (B) Cells from (A) were scored for their distribution as cytoplasmic or pancellular. (C) HeLa cells were transfected with GFP-SIRT2 and incubated with or without leptomycin B (LMB). (D) Cells from (C) were scored for localization of GFP-SIRT2 in the nucleus. (E) HeLa cells were transfected with GFP-SIRT2 followed by treatment with LMB. At indicated times, cells were fixed and visualized for nuclear localization.

Observation of HeLa cells transfected with an expression vector for SIRT2 tagged at the amino terminus with GFP revealed a minor fraction of cells with GFP-SIRT2 in both the nucleus and cytoplasm (Figure 1A); SIRT2 was exclusively cytoplasmic in 99.5% of cells and pancellular in 0.5% (Figure 1B). These data are consistent with biochemical and immunolocalization data demonstrating a minor fraction of endogenous SIRT2 localized in the nucleus [7], [21].

Since most of the GFP-SIRT2 was in the cytoplasm, we hypothesized that SIRT2 is either actively transported into the nucleus in a small fraction of cells or is actively exported from the nucleus in a majority of cells. To distinguish between these two models, we treated HeLa cells with leptomycin B (LMB), a specific inhibitor of the Crm1-dependent nuclear export pathway [27]–[30]. A substantial fraction of LMB-treated cells showed GFP-SIRT2 in the nucleus (Figure 1C): nuclear localization of SIRT2 was observed in 92.1% of LMB-treated cells and 7.9% of cells treated with a vehicle control (Figure 1D). These results indicate that SIRT2 is actively exported from the nucleus in a Crm1-dependent manner.

SIRT2 accumulated rapidly in the nucleus beginning at 30 min after the addition of LMB to cultures (Figure 1E). By 60 min, the amount of SIRT2 in the nucleus had increased dramatically and did not further increase with continued treatment for 240 min or 16 hrs. These import kinetics were observed in all cells in an asynchronously dividing culture. These observations therefore show that nuclear import and export do not depend on cells transiting through mitosis; therefore SIRT2 continuously shuttles between the nucleus and the cytoplasm.

### Identification of a NES in SIRT2

**Figure 2:**
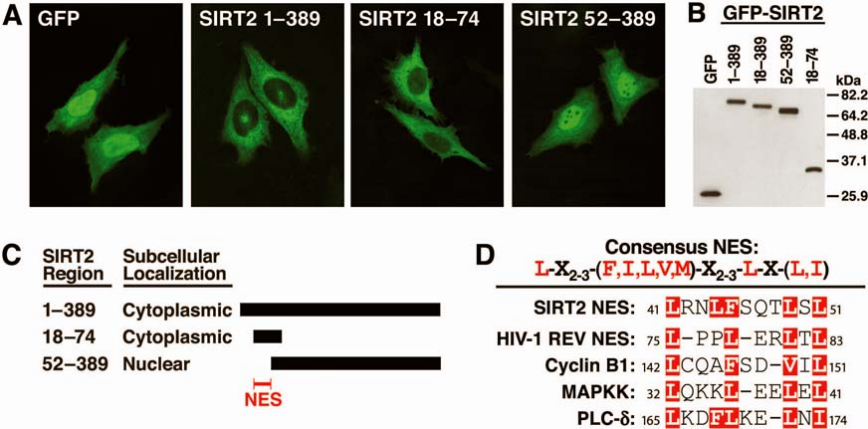
A Crm1-dependent NES is located in the amino-terminus of SIRT2. (A) HeLa cells were transfected with GFP or full-length and deletion mutants of GFP-SIRT2 and visualized for subcellular distribution. (B) 293T cells were transfected with cDNAs used in (A) and lysates were separated by SDS-PAGE and visualized by western blotting with an antiserum specific for GFP. (C) Schematic diagram of deletion analysis GFP-SIRT2 subcellular distribution. The region required for cytoplasmic localization is indicated. (D) Schematic of consensus Rev-like NES and proposed SIRT2 NES. Nuclear export signal sequences from proteins exported from the nucleus in a Crm1-dependent manner.

We next sought to identify the region of SIRT2 responsible for Crm1-dependent nuclear export with a series of SIRT2 deletion mutants fused to the carboxyl terminus of GFP. Fusion of SIRT2 amino acids 18–74 to GFP showed nuclear export of the fusion protein, while the fusion protein encompassing SIRT2 amino acids 52–389 showed pancellular distribution (Figure 2A). Generation of proper fusion proteins was confirmed by SDS-PAGE and western blot analysis (Figure 2B). These results demonstrate that the NES sequence of SIRT2 is found between amino acids 18 and 74 (Figure 2C).

Analysis of this region revealed a canonical Crm1-dependent NES sequence between amino acids 41–51 (Figure 2D). Comparison of this region to NES sequences from proteins previously known to shuttle in a Crm1-dependent manner [31]–[34], revealed a high degree of sequence similarity (Figure 2D).

**Figure 3:**
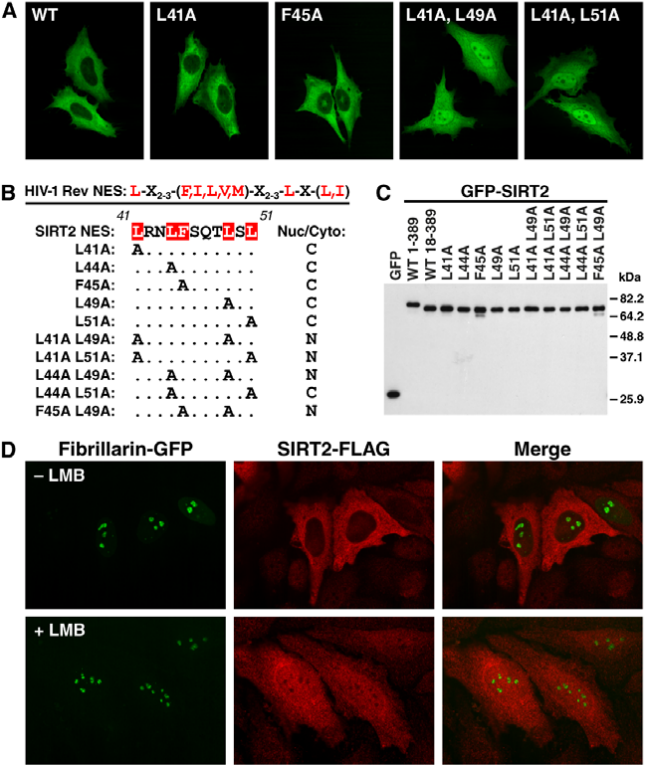
Mutational analysis of SIRT2 NES sequence. (A) HeLa cells were transfected with GFP-SIRT2 wild-type or single and double point mutants of the proposed NES sequence. (B) Subcellular distribution results of all single and double mutants of SIRT2 NES analyzed in (A). (C) 293T cells were transfected with GFP-SIRT2 single and double point mutants and lysates were separated by SDS–PAGE and visualized by western blotting with an antiserum specific for GFP. (D) HeLa cells were transfected with SIRT2-FLAG and fibrillarin-GFP, treated with or without LMB for 2 hrs and subsequently stained for FLAG and visualize by confocal microscopy.

To identify the specific amino acids involved in the SIRT2 nuclear export activity, we generated several single and double point mutants of the conserved residues within this region (Figure 3A and B). Alanine substitution of any of the conserved residues alone did not inhibit nuclear export. However, mutation of multiple sites (e.q., L41 and L49) abolished the nuclear export of the NES, with the exception of L44,51A, which remained exclusively cytoplasmic. These results demonstrate that SIRT2 shuttles between the nuclear and cytoplasmic compartment via a Crm1-dependent NES in its amino terminus.

Nuclear-targeted GFP-SIRT2 appeared to be excluded from nucleoli, a phenomenon not observed with GFP alone (Figure 3D). To confirm this observation, we transfected HeLa cells with the nucleolus-localized protein fibrillarin [24], [25], tagged at its carboxyl terminus with GFP, and FLAG-tagged SIRT2 (SIRT2-FLAG). Like GFP-SIRT2, SIRT2-FLAG was predominantly cytoplasmic, and fibrillarin-GFP was localized as expected to nucleoli (Figure 3D) [24], [25]. Upon LMB treatment, SIRT2-FLAG was sequestered in the nucleus but was excluded from the nucleoli, which were defined by the localization of fibrillarin-GFP (Figure 3D).

### Localization of SIRT2 During Mitosis

**Figure 4:**
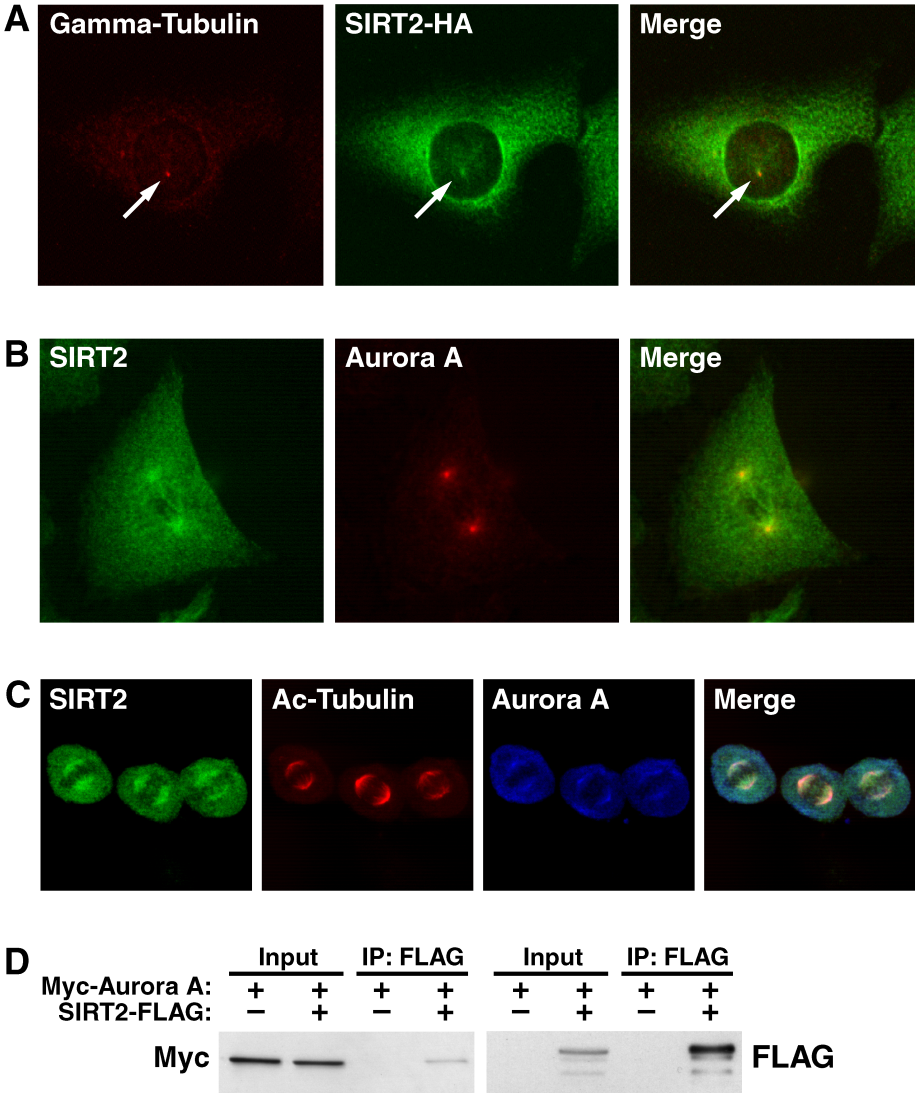
Colocalization of SIRT2 with the centrosome. (A) U2OS cells expressing SIRT2-HA were stained for HA (green) and for γ-tubulin (red) and analyzed by confocal microscopy. (B) HeLa cells were stained with antisera for endogenous SIRT2 (green) and Aurora A (red) and analyzed by confocal microscopy. (C) HeLa cells were stained with antisera for SIRT2 (green), acetylated tubulin (red), and Aurora A (blue) and analyzed by confocal microscopy. (D) 293T cells were transfected with Myc-Aurora A with or without SIRT2-FLAG. Cellular lysates were immunoprecipitated with anti-FLAG and probed by western blotting with antisera specific for FLAG and Myc. 10% of protein input was analyzed by western blotting with antisera for FLAG or Myc.

**Figure 5:**
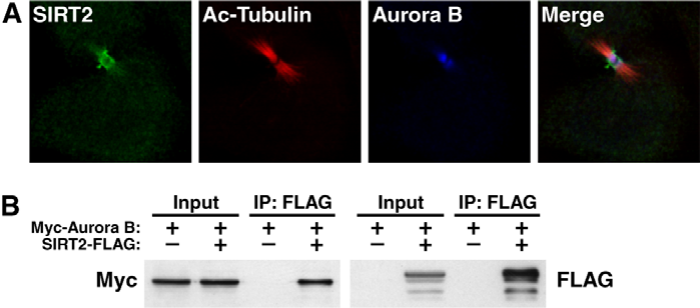
Enrichment of the three proteins on the midbody is shown in the merge images. (A) HeLa cells were stained with antisera for SIRT2 (green) and Aurora B (blue) and acetylated tubulin (red), and analyzed by confocal microscopy. Enrichment of the three proteins on the midbody is shown in the merged images. (B) 293T cells were transfected with Myc-Aurora A with or without SIRT2-FLAG. Cellular lysates were immunoprecipitated with anti-FLAG and probed by western blotting with antisera specific for FLAG and Myc. 10% of protein input was analyzed by western blotting with antisera for FLAG or Myc.

We observed in a fraction of cells in interphase had GFP-SIRT2 localized to a region similar to the centrosome. To confirm that SIRT2 was found localized to the centrosome in these cells, we transfected cells with SIRT2-HA and co-stained cells with antisera to HA and γ-tubulin, a marker of the centrosome (Figure 4A). During early prophase, endogenous SIRT2 became enriched at the centrosome demonstrated by its colocalization with Aurora A, a mitotic regulatory kinase also found on the centrosomes (Figure 4B and C) [35]. Utilizying coimmunoprecipitation experiments with FLAG-tagged SIRT2 and Myc-tagged Aurora A indicate that these two proteins interact in a mutliprotein complex (Figure 4D). At metaphase, SIRT2 remained concentrated in the centrioles and spread along the spindle fibers, consistent with Aurora A localization (Figure 5B). However, during cytokinesis, SIRT2 associated with the midbody, a structure formed by the bundled microtubules originating from polar microtubules after metaphase. We observed that SIRT2 colocalized with Aurora B, a midbody-localized protein [36] (Figure 5A). Colocalization of SIRT2 and Aurora B was further confirmed by the oberservation that both proteins interacted in co-immunoprecipitation experiments (Figure 5B).

### Overexpression of SIRT2 Increases Multinucleation

**Figure 6:**
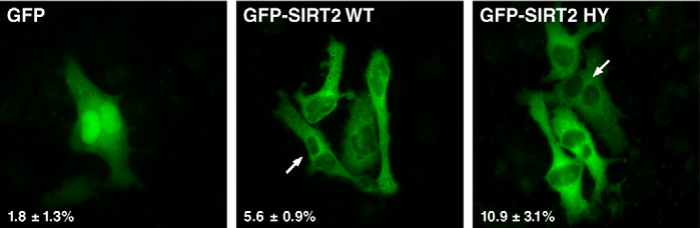
Overexpression of SIRT2 leads increases mutlinucleation. HeLa cells were transfected with GFP, and wild-type or H187Y GFP-SIRT2 and 48 hours after transfection, cells were scored for the presence of multiple nuclei. Percentages represent average of counting ∼200 cells in three independent transfections with error bars representing standard deviation.

Based on the selective enrichment of SIRT2 with mitotic structures, we hypothesized that overexpression of SIRT2 might affect the cell division. Introduction of mutations in proteins that are localized to the midbody often have subtle defects in cytokinesis, as was recently observed for CD2AP [37]. To determine if SIRT2 expression had adverse effects on cell division, we transfected cells with GFP or GFP-SIRT2 wild-type or a catalytically inactive GFP-SIRT2 mutant (H187Y). Two days after transfection, we scored cells containing two or more distinct nuclei indicating defective mitosis (Figure 6). Overexpression of wild-type GFP-SIRT2 increased by 3-fold, while overexpression of catalytically inactive GFP-SIRT2 resulted in a 5.5-fold in the number of cells containing 2 or more nuclei, suggesting that SIRT2 contributes to the proper progression through mitosis.

## Discussion

In this manuscript, we report that SIRT2 actively shuttles between the nucleus and cytoplasm and is excluded from the nucleus by a Crm1-dependent NES located in the amino terminus of SIRT2. Furthermore, the rate of nuclear export exceeds the rate of nuclear import, giving the appearance of constitutive localization of SIRT2 in the cytoplasm.

### Identification of a NES Sequence in SIRT2

A low percentage of cells transfected with an expression vector for SIRT2 showed both nuclear and cytoplasmic distribution. Treatment of asynchronously growing cells with the nuclear export inhibitor LMB resulted in accumulation of SIRT2 within the nucleus. LMB binds to the exportin Crm1 and inhibits it from recognizing NES sequences within cargo proteins. Under these conditions, SIRT2 entering into the nucleus is inhibited from binding to Crm1 and blocked from being exported leading to the nuclear sequestration of SIRT2. These results indicate that SIRT2 is actively exported from the nucleus and that its localization in the cytoplasm is mediated by a Crm1-dependent nuclear export signal. We were unable to identify a functional nuclear localization signal (NLS) within SIRT2, suggesting that SIRT2 is either imported by an undefined or multiple NLS sequences or piggy-backs onto another protein to mediate its import. The rapid kinetics of SIRT2 nuclear accumulation in response to LMB (within 60 min of treatment) indicates that SIRT2 constantly shuttles between the nucleus and cytoplasm during interphase.

The canonical NES recognized by Crm1 consist of a leucine-rich motif, similar to that initially characterized within the HIV-1 Rev protein. Deletion analysis of the GFP-tagged SIRT2 localized the NES of SIRT2 to amino acids 18–74. This SIRT2 domain contains a sequence that conforms to the Crm1-dependent NES consensus sequence, and mutagenesis studies confirmed its role in the nuclear export of SIRT2. Interestingly, neither LMB treatment nor mutation of the NES resulted in complete localization of SIRT2 in the nucleus, and a significant amount of the protein was maintained in the cytoplasm. These results indicate the potential of a second, LMB-insensitive export pathway in SIRT2 or the existence of a fraction of cytoplasmic SIRT2 that does not cycle between cytoplasm and nucleus, possibly via the binding of SIRT2 to a cytoplasmic structure.

### Enrichment of SIRT2 on Microtubule Structures During Mitosis

In addition to its controlled cytoplasmic localization during interphase, SIRT2 adopts a distinct localization pattern during mitosis. SIRT2 is phosphorylated in a mitosis-specific manner, indicating its potential role during this stage of the cell cycle [23]. We found that SIRT2 is enriched at the centrosome in prophase and on the growing spindle fibers throughout metaphase (colocalizing with both γ-tubulin and Aurora A). Colocalization of SIRT2 and γ-tubulin was not observed in every cell, suggesting that it might associate with the centrosome following centrosome maturation and as cells begin to transition from G2 into mitosis. As cells progress through telophase and into cytokinesis, SIRT2 is found on the midbody, where it colocalizes with Aurora B. These structures are primarily composed of microtubules, a *bona fide* substrate for SIRT2 [7], [38]. Unexpectedly, these microtubules appear to be hyperacetylated relative to cytoplasmic microtubules. This observation suggests that SIRT2 enzymatic activity may be regulated during mitosis, and that, although SIRT2 is found on these structures, it might be maintained in an enzymatically inactive state. Consistent with this hypothesis, overexpression of either wild-type or catalytically inactive SIRT2 results in defects in cytokinesis. These results suggest that SIRT2 activity is required for mitotic progression and may be temporally and spatially controlled to aid in the faithful completion of mitosis. However, at this time, our data demonstrating that overexpression of SIRT2 leading to an increase in multinucleated cells does not indicate where in the cell cycle SIRT2 could be regulating cell division that would lead to such a phenotype. Multinucleation can result from defects at a number of points in the cell cycle. For instance, cells which contain two perpendicular metaphase plates may attempt to divide into three cells in late anaphase followed by two of the three fusing in telophase, or cells may undergo failure to complete cytokinesis [39]. Based on our data, we are unable to determine if a function of SIRT2 at the centrosome, or the midbody, is responsible for regulating faithful completion of cell division. However, utilizing video microscopy, we have observed cell populations overexpressing GFP-SIRT2 containing a fraction of cells with two perpendicular metaphase plates. Cells with this chromosome arrangement during anaphase attempted to divide into three cells, but two of the three fuse in telophase, resulting in one cell with the appearance of one nuclei and the other appearing to have two nuclei (data not shown). Similar mitotic defects have reported in cells injected with anti-Aurora A antibodies [39]. Further studies addressing a role for SIRT2 in regulation of mitosis by Aurora A or B mitosis might uncover a mechanism for the observed multinucleation.

Sir2 proteins are involved in regulation of longevity in a wide variety of organisms [40]. Hst2p, a yeast homologue of Sir2p, can regulate yeast longevity in the absence of Sir2p by regulating *rDNA* stability [41]. Like SIRT2, Hst2p is predominantly localized in the cytoplasm, indicating that it likely shuttles into the nucleus to regulate nucleolar processes [15]. Consistant with this hypothesis, Hst2p was recently shown to contain a Crm1-dependent nuclear export signal [42]. Understanding the role of nuclear SIRT2 during interphase could potentially connect SIRT2 to the role of Sir2 proteins in aging.

The observation that SIRT2 is found on unique mitotic structures could indicate that it regulates the acetylation level of a cellular protein involved in the cell cycle, including tubulin. While tubulin is a recognized target of SIRT2 [7], [38], it is possible that SIRT2 deacetylates other proteins during the cell cycle. Finally, it is not clear what role SIRT2 plays in the nucleoplasm during interphase. Future studies will test the possibility that interphase nuclear SIRT2 might regulate the expression of a subset of genes or the acetylation level of other nuclear proteins.
